# SNP Mining in *Crassostrea gigas* EST Data: Transferability to Four Other *Crassostrea* Species, Phylogenetic Inferences and Outlier SNPs under Selection

**DOI:** 10.1371/journal.pone.0108256

**Published:** 2014-09-19

**Authors:** Xiaoxiao Zhong, Qi Li, Hong Yu, Lingfeng Kong

**Affiliations:** Key Laboratory of Mariculture Ministry of Education, Ocean University of China, Qingdao, China; Australian Museum, Australia

## Abstract

Oysters, with high levels of phenotypic plasticity and wide geographic distribution, are a challenging group for taxonomists and phylogenetics. Our study is intended to generate new EST-SNP markers and to evaluate their potential for cross-species utilization in phylogenetic study of the genus *Crassostrea*. In the study, 57 novel SNPs were developed from an EST database of *C. gigas* by the HRM (high-resolution melting) method. Transferability of 377 SNPs developed for *C. gigas* was examined on four other *Crassostrea* species: *C. sikamea*, *C. angulata*, *C. hongkongensis* and *C. ariakensis*. Among the 377 primer pairs tested, 311 (82.5%) primers showed amplification in *C. sikamea*, 353 (93.6%) in *C. angulata*, 254 (67.4%) in *C. hongkongensis* and 253 (67.1%) in *C. ariakensis*. A total of 214 SNPs were found to be transferable to all four species. Phylogenetic analyses showed that *C. hongkongensis* was a sister species of *C. ariakensis* and that this clade was sister to the clade containing *C. sikamea*, *C. angulata* and *C. gigas*. Within this clade, *C. gigas* and *C. angulata* had the closest relationship, with *C. sikamea* being the sister group. In addition, we detected eight SNPs as potentially being under selection by two outlier tests (fdist and hierarchical methods). The SNPs studied here should be useful for genetic diversity, comparative mapping and phylogenetic studies across species in *Crassostrea* and the candidate outlier SNPs are worth exploring in more detail regarding association genetics and functional studies.

## Introduction

Oysters are widely distributed throughout tropical and subtropical regions, inhabiting near-shore areas, shallow waters, bays, and estuaries [1]. *Crassostrea* oysters are important commercial species and account for most of the world's oyster production. Approximately 20 species make up the genus *Crassostrea*, of which *C. gigas* has become the leading species in world shellfish culture because of its rapid growth and capacity to adapt to various environmental conditions. Besides *C. gigas*, *C. hongkongensis*, *C. ariakensis*, *C. sikamea* and *C. angulata* are locally important species in China, Japan, Korea, the United States and some European countries. The rapid growth of the oyster aquaculture industry as well as intentional introduction or transplantation of oysters pressingly requires an appropriate understanding of the genetic variation within and among various oyster species. However, conventional taxonomic and phylogenetic studies based on morphology and geographic range information have proved problematic because of highly plastic shell patterns and overlapping geographic distributions [2]–[4]. There are ongoing debates as to the species designations in the genus *Crassostrea*, such as the specific status of *C. gigas* and *C. angulata*, and the nomenclature of *C. hongkongensis* and *C. ariakensis*. The ongoing confusion about oyster taxonomy and identification has become an impediment to further investigation of the genetics and conservation of oysters.

In recent years, relationships and identification of oyster species have been investigated by using allozymes, randomly amplified polymorphic DNA (RAPD), restriction fragment length polymorphism (RFLP) and DNA sequences such as mitochondrial and nuclear genes [5]–[11]. Particularly, the ability to sequence and compare whole mitochondrial genomes provides a new insight into phylogenetic relationships of oysters [12]–[14]. However, mtDNA loci are uniparentally inherited and cannot alone represent all historical and contemporary processes acting upon a population [15]. Moreover, because mtDNA is fast evolving and nucleotide mutations may return to an earlier state, its sequences may not allow deep phylogenetic reconstruction [12]. Hence, incorporating nuclear markers appears necessary to increase confidence in determining the relationships of *Crassostrea* species.

Single-nucleotide polymorphisms (SNPs) have become cornerstone markers for a wide variety of genetic applications because they are the most abundant class of polymorphisms in genomes, and can be genotyped cost-effectively [16], [17]. Besides, SNP can be found within the genomic sequences of gene candidates for artificial or natural selection and therefore they might be more informative for evolutionary biology than markers such as microsatellites and AFLPs. They offer a wide range of applications such as association studies, high-density linkage maps, traceability of genealogies and phylogenetic inference [18], [19].

The rapid increase in the availability of EST sequences of *Crassostrea gigas* provides abundant resources for obtaining SNP markers [20]–[23]. To date, 320 SNPs have been developed for *C. gigas* by mining expressed sequence tags data, using the HRM method [24]–[26]. Nevertheless, SNP markers for *C. hongkongensis*, *C. ariakensis*, *C. sikamea* and *C. angulata* have not been documented. Transferred SNPs from *C.gigas* provide a valuable source of SNP markers for the four species. Such cross-species EST–SNPs will be useful for comparative mapping and phylogenetic studies among species in *Crassostrea*.

Here, 57 novel SNPs were developed from the NCBI EST database (http://www.ncbi.nlm.nih.gov/) of *C. gigas* and the cross-species transferability of 377 SNPs of *C. gigas* was tested among *C. hongkongensis*, *C. ariakensis*, *C. sikamea* and *C. angulata*. Meanwhile, through the use of the cross-species SNPs, we reconstructed the phylogenetic relationships among the five *Crassostrea* species. Moreover, through the use of Fst outlier analysis, we identified candidate SNPs that may have been targets of selection.

## Materials and Methods

### Ethics Statement

The field studies did not involve endangered or protected species. No specific permissions were required for the locations. The locations are not privately-owned or protected in any way.

### Oyster Materials and DNA Extraction

Thirty-two *C. gigas* individuals from 2 populations (Pop1: 16 individuals from Weihai, Shandong province, China; Pop2: 16 individuals from Rizhao, Shandong province, China) were used for validation of SNP polymorphisms. Five *Crassostrea* species collected from China were used for the examination of the transferability of SNPs, namely *C. sikamea* (from Nantong, Jiangsu Province), *C. angulata* (from Yueqing, Zhejiang Province), *C. hongkongensis* (from Xiamen, Fujian Province), *C. ariakensis* (from Shantou, Guangdong Province) and *C. gigas* (from Rushan, Shandong Province) (Table 1). A set of species-specific COI primers was used for species identification according to the study of Wang & Guo [10].

**Table 1:** Species included in this study, and the statistics of amplification success and polymorphism.

Species	Number of individuals	Sample location (latitude, longitude)	Number	Percent	Number	Percent
			amplified	amplified	polymorphic	polymorphic
*C. sikamea*	20	Nantong, Jiangsu (31.91°N, 121.88°E)	311	82.50	256	67.90
*C. angulata*	19	Yueqing, Zhejiang (28.15°N, 121.08°E)	353	93.60	306	81.20
*C. hongkongensis*	19	Xiamen, Fujian (24.43°N,118.15°E)	254	67.40	133	35.30
*C. ariakensis*	19	Shantou, Guangdong (23.35°N,116.63°E)	253	67.10	119	31.60
*C. gigas*	19	Rushan, Shandong (36.90°N, 121.80°E)	377	100	335	88.90

DNA was extracted from frozen adductor muscle tissue by a modification of the standard phenol–chloroform procedure previously described by Li et al. [27] and stored at −30°C prior to genetic analysis.

### Data Mining for SNP Markers

Sequences containing SNPs were annotated using BLASTx software [28], and sequence homology was accepted based on a cut-off E value of 1.0×10^−6^. The informative strand and reading frame were identified by using the sequence with highest homology. The NCBI ORF finder (http://www.ncbi.nlm.nih.gov/gorf/gorf.html) was used to determine whether SNPs were synonymous, non-synonymous or from untranslated regions (UTRs).

### Primer Design and PCR Conditions

Primers were designed using the Primer Premier 5.0 program (PREMIER Biosoft International, Palo Alto, CA, USA). SNP markers were developed according to the procedure described by Zhong et al. [25] and genotyped using the high resolution melting (HRM) method on the LightCycler 480 real-time PCR instrument (Roche Diagnostics, Burgess Hill, UK). A total of 46,171 Pacific oyster EST sequences were downloaded from GenBank EST database (http://www.ncbi.nlm.nih.gov/). The sequences were assembled and clustered into contigs with SeqMan Pro software (DNASTAR Inc., Madison, WI, USA). A single-base mutation that occurred in four or more ESTs and that was surrounded by good flanking sequences was identified as a potential SNP for further analysis.

The 10-µl reaction mixture contained 0.25 U Taq DNA polymerase (Takara, Dalian, China), 10× PCR buffer, 0.2 mM dNTP mix, 0.2 µM of each primer set, 1.5 mM MgCl_2_, 5 µM SYTO9 (Invitrogen Foster City, CA, USA) and 10 ng template DNA. The concentration of DNA was measured by a Nanodrop 2000 spectrophotometer (Thermo Scientific, Waltham, MA). The PCR cycling conditions included an activation step at 95°C for 5 minutes followed by 45–50 cycles of 95°C for 20 seconds, a touch down of 68°C to 58°C for 20 seconds (0.5°C/cycle) and 72°C for 20 seconds. Following amplification, the products were denatured at 95°C for 1 min, and then annealed at 40°C for 1 min to randomly form DNA duplexes. Melting curves were generated by heating samples from 60°C to 90°C with 25 data acquisitions per degree. Data were analyzed using the LightCycler 480 Gene Scanning Software 1.5 (Roche Diagnostics).

### Data Analysis

Shannon's Information index, expected heterozygosity (*H_e_*), observed heterozygosity (*H_o_*) and Nei's genetic distance [29] were calculated using POPGENE 1.32 software [30]. Phylogenetic trees were constructed using the neighbor joining (NJ) method implemented in MEGA 5.05 and POPTREE2 [31], [32]. Bootstrap analyses with 1000 replicates were performed to test the support for the branches of a phylogenetic tree.

Arlequin version 3.5.1.3 software was used to calculate pairwise Fst between all pairs of species using 10000 permutations to test for significance (0.01). Outlier SNPs were tested using two island models, as implemented in Arlequin. We conducted 50000 coalescent simulations with 5 demes under a finite island-model. The analysis was also performed utilizing a hierarchical island model based on 3 groups of 3 demes with 50000 simulations to generate the joint distribution of Fst versus heterozygosity. Pre-defined population groupings were set as three groups (group 1: *C. sikamea, C. angulata* and *C. gigas*; group 2: *C. hongkongensis*; group 3: *C. ariakensis*) based on the pairwise Fst values. Loci that fall out of the 99% confidence intervals of the distribution were identified as outliers being putatively under selection. The putative function of genes with outlier SNPs was identified using the Gene Ontology (GO) annotation by mining the Swiss-Prot database.

## Results

### Development and Transferability of SNPs

In the study, 262 putative SNPs were selected for validation. Among these, 57 SNPs (22%) were polymorphic and considered as validated. Information about the panel of loci is summarized in Table 2. The 57 substitutions included 41 transitions and 16 transversions. Of the polymorphic SNPs, 30 (52.6%) could not be annotated, 53 (93.0%) were located in the coding region, and 4 (7.0%) in the UTR. Eighteen of the 53 SNPs located within the coding region were nonsynonymous and 35 synonymous.

**Table 2:** Characterization of 57 polymorphic EST–SNPs derived from *Crassostrea gigas*.

SNP name	Accession no.	Primer sequences (5′-3′)	Amplicon length (bp)	SNP type and location	Type	Annotation
CgSNP879	HS148847	F: ACTGGTCTCACCCCCATCAC	60	C/T (487)	S (Pro)	Unknown
		R: AGTCCTATTCACTTCACTGCTGC				
CgSNP880	HS140594	F: AAGTGGTCATCGAAAAAGGTCTTC	90	G/T (632)	S (Leu)	Glutathione S-transferase theta-1
		R: CGGCGAGGTATTTAGACTTCTCC				
CgSNP882	HS243771	F: TTAGAACCGATAATCCAAGGAAGTC	76	A/G (209)	S (Glu)	ADP-ribosylation factor-like protein 15
		R: ACAATCATCTTTACTATTTTCTCTGCC				
CgSNP886	HS210205	F: TCTGGAAATACAATCTGCTGGC	71	C/T (221)	S (Asp)	hypothetical protein CGI_10018860
		R: CCTGGCTTTGATGAGGGCTT				
CgSNP890	HS236510	F: CGGAGTCGAATGAAACAGGAT	77	A/G (112)	S (Pro)	Unknown
		R: TAGGTCTGATACATTGAAGTAAGCG				
CgSNP891	HS236510	F: TCTACATCGAAGGACAATTTTCAAG	70	G/T (250)	N (Ser-Arg)	Unknown
		R: TTCCCGTTTCGGATATACAGACT				
CgSNP895	FP008693	F: CTCGGTCTCAGTCATTGCGG	67	A/G (82)	S (Met)	Unknown
		R: GATTTCTCCTCTATCCTGCTTTCC				
CgSNP900	HS238336	F: TCCTGATAACATTGCTGTGTTTG	70	A/C (166)	S (Gly)	Protein BAT5
		R: GTAGTTCATTGCTACCCATGATGC				
CgSNP909	CU682103	F: TTACAATTCAGAACAGGACAATGG	74	A/T (207)	S (Leu)	Macrophage mannose receptor 1
		R: ACAAACTTTGAGTCTATGACTCGGT				
CgSNP913	HS167108	F: TGTTGGGAACGATTCATACGG	77	C/T (271)	S (Asp)	hypothetical protein CGI_10025728
		R: CATTTCGGTGTTCACGATTGG				
CgSNP915	FQ661219	F: CCAATCCAGTGCCAAAGTCTC	80	A/G (317)	S (Glu)	Unknown
		R: CAGCAACTAAATGGTCCACATAAC				
CgSNP917	HS175405	F: TTGTCCTTGTTAATTACTGCATTGC	70	C/T (226)	S (Cys)	Unknown
		R: GCCTAGTTTGCGTAGGAGAGAG				
CgSNP924	HS175248	F: GCGGAGTCGGAGCATCAG	58	C/T (261)	S (Cys)	ELKS/RAB6-interacting/CAST family member 1
		R: TCAGGTCGTGGTTCCTCTTCAT				
CgSNP936	CU993732	F: CACACAAGAAGAAAACGCACAAGAT	86	A/G (604)	S (Glu)	Phosphatidylinositol-3,4,5-trisphosphate 3-phosphatase TPTE2
		R: TGGTAAAAGATGTCAGGAACAAGGT				
CgSNP940	HS223847	F: ATCACGACTGTAGGGCAGAGATTAT	81	G/T (202)	N (Gln-His)	Unknown
		R: AGGTTTGGATTGAGCTTTTGTCTAG				
CgSNP942	HS191752	F: CCTCGGATCTGTTGATTGCTATT	72	A/G (564)	S (Pro)	Complement C1q tumor necrosis factor-related protein 3
		R: TGTTCTGCCAGGGTATGTTCG				
CgSNP949	HS231194	F: CATCTCAGGGAAATGGAAGG	72	C/T (491)	S (Tyr)	Tetratricopeptide repeat protein 17
		R: AAGAAACAAAATAATGAAGAGCG				
CgSNP958	HS109673	F: AATCCTTGATGAGCCGACG	82	C/T (715)	S (Ala)	ATP-binding cassette sub-family F member 3
		R: CCCTCCCTGGAATTTCAGTAT				
CgSNP970	HS206217	F: AAGAGATTTTATTGTAGAAGTTGACATAT	88	G/T (125)	S (Ser)	Unknown
		R: CATACCAAAAGAATCAATGAATACTC				
CgSNP980	HS201459	F: AAGACTGTGTGACGGTTCAGATG	82	A/G (471)	S (Ser)	Unknown
		R: AGCAGTGAAATGTTGGCGAT				
CgSNP989	HS242001	F: GCAGTGCATGTGGATGAGTAAGT	81	G/T (184)	UTR	Unknown
		R: CGCCATAAAGTTGAAAGTATTGAAC				
CgSNP990	HS227296	F: GGTTCCATTAAGCCATCCATTG	71	C/T (586)	UTR	Unknown
		R: GCAGACAGTATCAGCAGTCGTTG				
CgSNP994	HS227373	F: TGTATTTCAAGGCGTGTTACAGTG	84	C/T (694)	N (Cys-Arg)	Unknown
		R: ACTCATCAGTCAAGGGACAACAAG				
CgSNP1003	CU996515	F: GTGAGAGACTGATGAGTGCCTGT	72	C/T (529)	S (Gly)	Unknown
		R: TATGAGTGATCAGGAATTCTGTAGC				
CgSNP1010	FQ668992	F: TCAAATCAAATCTGAACGGCG	74	C/T (580)	S (Gly)	Fibrinogen C domain-containing protein 1
		R: CCAGTTATTGTACGGTCCCCAT				
CgSNP1016	CU997800	F: ATGTGATTGTCTCTTGAGAATGTGT	75	C/T (588)	N (Val-Ala)	Unknown
		R: CAGAGATGAAACCAGTATGTCTGAT				
CgSNP1019	HS116482	F: TCAGACACGGAGGGAAAATG	98	C/T (430)	S (Pro)	39S ribosomal protein L45, mitochondrial
		R: TCTTTGTCCTCTTTCCAAGTGTG				
CgSNP1021	HS122227	F: AGCCCACTGGAGGAAGAACC	58	C/T (154)	N (Val-Ala)	Unknown
		R: GGTATTCGGGATTGAATCTGTG				
CgSNP1023	HS235875	F: GCACTACATATCATACCAGACTGTG	104	A/G (377)	N (Asn-Asp)	Putative arylformamidase
		R: GTTTGTAAAATAATGCCCATAACTG				
CgSNP1024	FQ666947	F: GTCTAGGAGTTATTTCCCTTTGATG	98	A/C (554)	UTR	Unknown
		R: TGGATTTAGTGTTCACCAGTACAAG				
CgSNP1028	CU997294	F: ACAGACAAAATGACAAGAAAACAAC	76	C/T (606)	S (Ser)	Unknown
		R: CAGTGACCTCAGCAGCCATC				
CgSNP1029	CU997294	F: CTCTCACACCAGATATTTCCAGCAT	82	A/G (630)	N (Lys-Glu)	Unknown
		R: CTTCCTTTCAAGGTCACAATCACAC				
CgSNP1034	HS180370	F: CCTGTCTTTTAACACTGTTTCTGAT	97	A/T (322)	N (Trp-Ser)	Unknown
		R: GTCAGGACGTTTTCTGCTTTC				
CgSNP1037	HS248681	F: CCAAAGTGTACGCTGTAAGGAACC	81	G/T (161)	S (Ser)	Unknown
		R: CGTCAATGCTGATGGACAAGG				
CgSNP1038	HS239387	F: GCTATACCTTGTCCATCAGCATTG	60	C/T (708)	N (Lys-Glu)	Ufm1-specific protease 1
		R: CATTAGTGTTGTTCACGGGGAG				
CgSNP1042	HS229886	F: AAGTCAGTGAAGAGCCACAAAC	84	A/C (280)	S (Ser)	Interleukin-1 receptor-associated kinase 1
		R: AAACCTCATTAAATCCCAAGTGT				
CgSNP1043	FP004709	F: CAAGTTCCGAATGAAATACCTTCT	85	C/T (554)	N (Tyr-Cys)	hypothetical protein CGI_10008375
		R: CTCAAAATAGCTGTCCCTGTGTG				
CgSNP1045	FP004709	F: GACAGATAACAACTCTCAAGCAAAC	67	A/C (688)	S (Leu)	hypothetical protein CGI_10008375
		R: CACATATCGTTACGAAACCGAG				
CgSNP1047	HS233108	F: TCTGGAGGCTGTATGCTGAGTT	65	A/G (364)	S (Gln)	Tetratricopeptide repeat protein 27
		R: CTTTTGTTGTGTTTCCGCTGT				
CgSNP1050	CU986514	F: CAAGTGTCCTGTATGTTGACAGTC	64	A/G (754)	N (Met-Val)	Mu-crystallin-like protein
		R: GATAAAATTACATCCCCACTCTCTT				
CgSNP1052	HS170919	F: TCCTGTTGCATCAGTATTCAAGATT	87	A/T (233)	N (Leu-Ter)	Unknown
		R: AAGCCTCAAAGTATGACCAGCAC				
CgSNP1054	FP010213	F: GTAGCTTGGATATTACTGTGAGGC	77	G/T (205)	UTR	Unknown
		R: CATGGAAATCTCGGTATAAACTTG				
CgSNP1055	FP010213	F: GATGAGTGCTTACATCAATCTGAGT	92	C/T (371)	N (Met-Thr)	Unknown
		R: CAAGACACAAAAACACATGCTTATAC				
CgSNP1056	HS162699	F: GCTGTTTGGTCTGGTGTTTGT	79	A/G (567)	N (Asn-Asp)	Unknown
		R: TTGAAAGCATGAAGATTTCTATCAC				
CgSNP1058	CU997792	F: AAGGAAATTCCCTGCACAAAC	78	A/C (967)	N (Ile-Leu)	GTP-binding protein GEM
		R: GTCCACACAAGATAAAAGAGAAGAG				
CgSNP1061	CU986467	F: CAGAGGACCAGTTTGAGGCTT	63	A/G (798)	S (Val)	BCCIP-like protein
		R: CTTGTTTGAGTTTGTCTGCGG				
CgSNP1069	HS137887	F: CGTGGAAATTCTGTGTAAATAGGAC	76	C/T (377)	N (Ser-Gly)	Unknown
		R: CTTCGGTTCGATTATGCTGC				
CgSNP1073	HS139503	F: GCTGCCAGTTTTTCTCATTCAC	74	C/T (523)	S (Thr)	15-hydroxyprostaglandin dehydrogenase [NAD+]
		R: AACCAAGGACACATACGGACAAC				
CgSNP1074	HS220139	F: CATGGTGACTAAATCTTCAATGTTGT	87	A/G (465)	S (Ser)	Exosome component 10
		R: AAGGCTGTGAGTAGAGGTTTGGC				
CgSNP1077	AM853850	F: CTGAGGCACAAAGTCTGGGTAGT	73	C/T (356)	N (Met-Thr)	Unknown
		R: GGAGGAGTAGGTGACCGCTTC				
CgSNP1082	FP009397	F: TATTAGGACCACATTCAGCTATGTC	88	A/G (256)	S (Pro)	Unknown
		R: ATTGATGGGGGTGGAGGTAC				
CgSNP1105	FP008773	F: CAAGAGTTGACACCAGAGGGAG	83	C/T (285)	S (Thr)	Unknown
		R: CATCAAATACACGATGACCTGAG				
CgSNP1115	HS204076	F: TCGGTCACTGTTGGATTTCTG	84	A/G (378)	S (Leu)	Heat shock 70 kDa protein 12B
		R: GAACAACCCGAATTCACGACC				
CgSNP1117	HS116629	F: TAGTAAAGGCTAAACAAAGTGTGCT	71	G/T (337)	S (Val)	Unknown
		R: AGGGAGAGTCCGAGATGTCAC				
CgSNP1118	CU998279	F: GACGAGTGAACGAGTACGGC	65	C/T (198)	S (Tyr)	Protocadherin-19
		R: TGGTCTATACGCAGAATAAGGAAT				
CgSNP1130	HS142312	F: CAAGGGACAGAGTTCAATGTCTTCT	86	A/G (585)	S (Leu)	Unknown
		R: TGACAGGATTTCTTGCATCTTTACC				
CgSNP1131	HS225071	F: ATGTGCTTTTTACCCGAACTGC	63	A/G (477)	N (Asp-Asn)	Poly [ADP-ribose] polymerase 12
		R: ACCTGTTTTGGTTGCTCGTCTT				

Note: S, synonymous; N, non-synonymous; UTR, untranslated region.

A total of 377 SNPs of *C. gigas* including 320 previously developed SNPs [24]–[26] and 57 new SNPs developed here were used to test the transferability in 4 other *Crassostrea* species: *C. sikamea*, *C. angulata*, *C. hongkongensis* and *C. ariakensis*. The basic information obtained with each SNP is shown in Table S1. Out of the 377 primer pairs tested, 311 (82.5%) primers showed amplification in *C. sikamea*, 353 (93.6%) in *C. angulata*, 254 (67.4%) in *C. hongkongensis*, 253 (67.1%) in *C. ariakensis* and 377 (100%) in *C. gigas*. Using the 377 primer pairs, 256 (67.9%) SNP loci were polymorphic in *C. sikamea*, 306 (81.2%) in *C. angulata*, 133 (35.3%) in *C. hongkongensis*, 119 (31.6%) in *C. ariakensis and* 335(88.9%) in *C. gigas* (Table 1). In total, 214 SNPs could give successful amplification in all the five *Crassostrea* species and 48 SNPs showed polymorphism in all the five species.

### Phylogenetic Relationships

**Figure 1:**
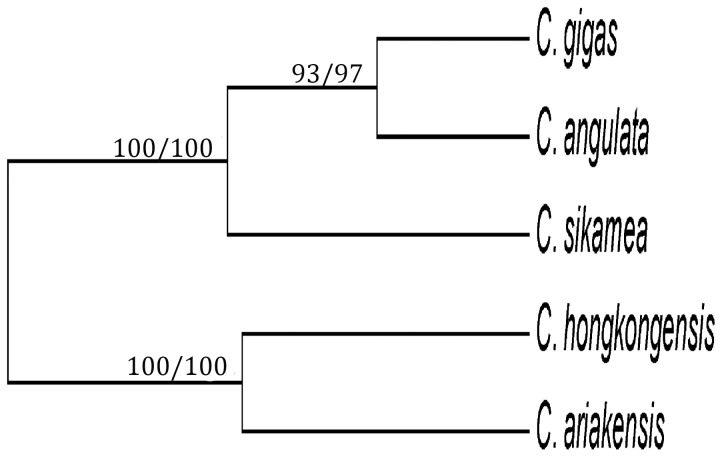
Phylogenetic tree of five *Crassostrea* species using neighbor joining (NJ) method based on Nei's genetic distance derived from 214 SNPs. Numbers above branches indicate bootstrap values from NJ analysis using both MEGA 5.05 and POPTREE2 softwares.

A total of 214 SNPs was used for the phylogenetic analysis. Information of the 214 SNPs evaluated from the 5 species is shown in Table 3. The values of observed heterozygosity (*Ho*) and expected heterozygosity (*He*) ranged from 0.0792 (*C. hongkongensis*) to 0.2895 (*C. gigas*) and from 0.1026 (*C. hongkongensis*) to 0.3229 (*C. gigas*), respectively. Shannon's Information index and the number of polymorphic loci ranged from 0.1664 (*C. ariakensis*) to 0.4749 (*C. gigas*) and from 99 (*C. ariakensis*) to 201 (*C. gigas*). Nei's genetic distance values ranged from 0.0738 (*C. angulata* and *C. gigas*) to 0.2728 (*C. hongkongensis* and *C. gigas*) (Table 4). All Fst estimates were statistically significant (*P*<0.01). Pairwise Fst ranged from 0.1230 (*C. angulata* and *C. gigas*) to 0.5257 (*C. hongkongensis* and *C. ariakensis*). The phylogenetic tree separated the five species into two clusters (Figure 1). The first cluster included two species, *C. hongkongensis* and *C. ariakensis*. This clade was sister to the clade containing *C. sikamea*, *C. angulata* and *C. gigas*. In this clade, *C. gigas* and *C. angulata* had the closest relationship, with *C. sikamea* being the sister group. Phylogenetic analysis using the unweighted pair-group method with arithmetic mean (UPGMA) generated an identical topology with high support values (data not shown).

**Table 3:** Characterization of 214 polymorphic EST-SNPs evaluated from 5 *Crassostrea* species.

Species	SI	*Ho*	*He*	Number polymorphic	Percent polymorphic
*C. sikamea*	0.3617±0.2442	0.1942±0.1792	0.2394±0.1827	176	82.24
*C. angulata*	0.4196±0.2325	0.2538±0.1924	0.2829±0.1763	186	86.92
*C. hongkongensis*	0.1691±0.2040	0.0792±0.1197	0.1026±0.1383	111	51.87
*C. ariakensis*	0.1664±0.2166	0.1021±0.1627	0.1039±0.1469	99	46.26
*C. gigas*	0.4749±0.2000	0.2895±0.1860	0.3229±0.1569	201	93.93

Note: SI, Shannon's Information index; *He*, expected heterozygosity; *Ho*, observed heterozygosity.

**Table 4:** Pairwise Nei's genetic distance (lower diagonal) and Fst values (upper diagona) among 5 *Crassostrea* species using 214 SNPs.

Species	*C. sikamea*	*C. angulata*	*C. hongkongensis*	*C. ariakensis*	*C. gigas*
*C. sikamea*		0.2486	0.5250	0.5045	0.2662
*C. angulata*	0.1327		0.4827	0.4824	0.1230
*C. hongkongensis*	0.2641	0.2525		0.5257	0.4707
*C. ariakensis*	0.2420	0.2535	0.1396		0.4402
*C. gigas*	0.1607	0.0738	0.2728	0.2380	

### Outlier SNPs

**Figure 2:**
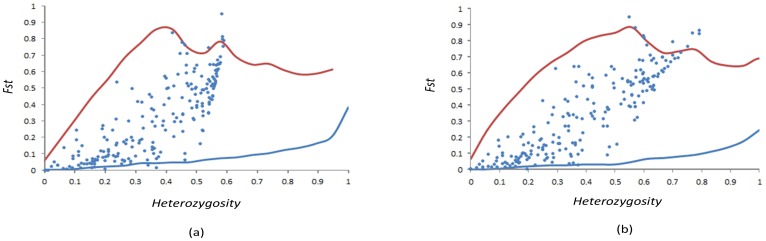
Plot of Fst against heterozygosity for 214 SNPs analysed with the fdist (a) and hierarchical (b) methods. The upper and lower lines are the 99% confidence intervals.

Loci showing higher or lower differentiation with respect to the simulated confidence intervals are identified as candidates for positive or balancing selection [33]. The Arlequin fdist method revealed 10 candidate SNPs (CgSNP28, CgSNP230, CgSNP273, CgSNP415, CgSNP420, CgSNP515, CgSNP524, CgSNP544, CgSNP669 and CgSNP805) for selection, including 7 for positive selection and 3 for balancing selection (Table 5 and Figure 2a). In addition, the hierarchical method detected 11 outlier loci (CgSNP14, CgSNP203, CgSNP803, CgSNP273, CgSNP415, CgSNP420, CgSNP515, CgSNP524, CgSNP544, CgSNP669 and CgSNP805) for selection, including 9 for positive selection and 2 for balancing selection (Table 5 and Figure 2b). Both approaches revealed 8 SNPs lying outside the 99% confidence region of the conditional joint distribution of Fst and heterozygosity, including 6 for positive selection and 2 for balancing selection. Among the 8 SNPs, 5 located within the coding region were synonymous and 3 nonsynonymous. The putative function of three genes (UPF0686 protein, ankyrin repeat domain-containing protein 60, and hypothetical protein CGI_10016494) could not be identified using GO searches. The other five proteins (endoglucanase, rho-related GTP-binding protein, flap endonuclease 1-A, hypothetical protein CGI_10023940 and Chlorophyllase-2) were respectively involved in carbohydrate metabolism, GTPase-mediated signal transduction, DNA repair, DNA binding and chlorophyll catabolic process.

**Table 5:** Outlier SNPs detected using the finite island model and hierarchical island model for Fst calculation.

	Finite island model			Hierarchical island model				
Locus	*Ho*	Fst	*P*	*Ho*	Fst	*P*	Type	Annotation
CgSNP28	0.5384	0.7438	0.0063	―	―	―	S (Asn)	Pannexin3
CgSNP230*	0.3396	0.0297	0.0047	―	―	―	N (Ser/Glu)	Unknown
CgSNP14	―	―	―	0.5687	0.8787	0.0090	N (Pro/Leu)	Unknown
CgSNP203	―	―	―	0.7503	0.7636	0.0089	N (Arg/Cys)	Adenylate kinase 2, mitochondrial-like
CgSNP803	―	―	―	0.6996	0.7928	0.0028	S (Ala)	Eukaryotic translation initiation factor 6
CgSNP273	0.4505	0.7763	0.0140	0.5985	0.8316	0.0100	S (Val)	UPF0686 protein C11or f1-like protein
CgSNP415	0.5893	0.7905	0.0079	0.7922	0.8442	0.0024	S (Ala)	Endoglucanase
CgSNP420*	0.1962	0.0085	0.0023	0.1947	0.0011	0.0003	S (Ala)	Rho-related GTP-binding protein RhoU
CgSNP515	0.4607	0.7619	0.0117	0.6008	0.8174	0.0116	N (Ile-Met)	Flap endonuclease 1-A
CgSNP524	0.5822	0.9492	0.0000	0.5492	0.9462	0.0035	S (Arg)	Ankyrin repeat domain-containing protein 60
CgSNP544	0.5825	0.7967	0.0070	0.7682	0.8459	0.0021	S (Ser)	Hypothetical protein CGI_10023940
CgSNP669*	0.3672	0.0135	0.0014	0.3717	0.0254	0.0063	N (Asp-Gly)	Chlorophyllase-2
CgSNP805	0.5832	0.8139	0.0046	0.7913	0.8628	0.0016	N (Ser-Ala)	Hypothetical protein CGI_10016494

Note:

* balancing selection; *Ho*, observed heterozygosity.

## Discussion

A total of 48769 potential SNPs were detected by mining the *C. gigas* EST database [25]. In our studies, the 1283 putative SNPs selected for validation allowed the development of 57 new SNPs bringing the total to 377 SNPs that have been validated in this species [24]–[26],. Among the 377 SNPs, 66 SNPs are known to be distributed in 8 linkage groups of *C. gigas* [26]. Compared to the use of several (often partial) genes, the adequate number of EST–SNPs, distributed in almost all linkage groups of *C. gigas*, may provide more genetic information which is valuable for phylogenetic analyses. The high cross-species transferability of the set of 377 EST-SNPs of *C. gigas* tested in four other *Crassostrea* species also suggests their potential utilization in evolutionary analysis across taxa of the genus *Crassostrea*. Moreover, mutations resulting in some SNPs can be responsible for an adaptive phenotype or the direct target of selection. Studies have shown that variation in allele frequencies at some outlier SNP loci can be correlated with environmental variables, such as salinity and temperature [34], [35]. Consequently, the SNP markers offer a valuable opportunity to understand the genetic basis of phenotypic variation in relation to environmental variation.

In general, the more evolutionarily distant the taxa, the less successful is cross amplification [36], [37]. In a previous study, 15 EST-SSRs developed for *C. gigas* amplified successfully in at least one species, with *C. sikamea* sharing 14 (93.3%) primer pairs, *C. hongkongensis* 12 (80.0%), and *C. ariakensis* 11(73.3%) [38]. Hedgecock et al. [39] tested 86 genomic SSRs developed for *C. gigas* in cross-species amplification, 83 (96.5%) were likely useful for *C. angulata*, 71 (82.6%) for *C. sikamea* and 31 (36.0%) for *C. ariakensis*. Our data also showed *C. angulata* (93.6%) and *C. sikamea* (82.5%) had higher cross-amplification rates than both *C. hongkongensis* (67.4%) and *C. ariakensis* (67.1%). These results suggest that *C. gigas* has a closer relationship with *C. angulata* and *C. sikamea* than with *C. hongkongensis* and *C. ariakensis*.

The taxonomy of *Crassostrea* has been studied for many years, but confusions still exist. There is an open debate as to whether *C. gigas* and *C. angulata* are distinct species [9], [13], [40], [41]. Some experts have argued that they are different species but genetically closely related [12], [40], [41], but other phylogenetic analyses suggest that the two should be considered one species [9], [42]. In our study, *C. gigas* and *C. angulata* were recovered as separate clades, suggesting that *C. gigas* and *C. angulata* may be two distinct species. However, the low Nei's genetic distance value between *C. angulata* and *C. gigas* (0.0738) indicates a very close relationship between them. Furthermore, *C. angulata* and *C. gigas* can cross-fertilize without any difficulty in the laboratory and form viable, fertile offspring [43]–[45]. Therefore, we still can not conclude that *C. gigas* and *C. angulata* are two distinct species. A large amount of the two species sampled from a wide geographic range and the same locations are required to better resolve this problem. Another species, *C. hongkongensis* has been routinely misidentified as *C. ariakensis* for a long time. In our study, *C. hongkongensis* and *C. ariakensis* were recovered as separate clades. Moreover, the Nei's genetic distance between *C. hongkongensis* and *C. ariakensis* (0.1396) was a little higher than that observed between two closely related sister species (between *C. angulata* and *C. sikamea*, 0.1327). The above data suggest that *C. hongkongensis* and *C. ariakensis* are two distinct species. Yu & Li [14] analyzed the complete mitochondrial DNA sequence and determined that *C. hongkongensis* and *C. ariakensis* are two separate species. Reece et al. [9] also suggested that the *C. ariakensis* sequences formed a distinct clade from *C. hongkongensis* in the COI tree. Therefore, we can conclude that *C. hongkongensis* and *C. ariakensis* are two separate species.

Identifying the regions of the genome that are shaped by adaptation to different environments can be relevant to answering several important questions in evolutionary biology. Among many selection detection strategies, Fst outlier approaches are becoming widely used in identifying genes without known phenotypes that are under selection [33], [46], [47]. These methods can identify relatively highly differentiated markers (so-called outlier loci) in comparison to expected levels under neutrality inferred from coalescent simulations [48], [49]. Strong outlier patterns have been classically interpreted as being caused by divergent selection affecting the loci themselves or genes strongly linked with them [50]. Indeed, an alternative explanation for strong genetic divergence at some loci exists and is difficult to rule out when the tests are being made on comparisons of distinct species. Bierne et al. [51] advocate the role of pre- or postzygotic genetic barriers in genetic divergence. Such endogenous barriers could be the consequence of incompatibilities between combinations of alleles, established through selective mechanisms that are independent from adaptation to habitats [35]. To increase confidence in the conclusions reached, two-island models and a high confidence level (99%) were used in the Fst outlier analysis.

Eight loci were identified as being possible targets of selection following two Fst outlier tests. Among the 8 SNPs, 5 located within the coding region were synonymous and 3 nonsynonymous. While nonsynonymous outlier SNPs are particularly interesting due to the potential effect of amino acid changes on protein structure and function, synonymous SNPs should not be simply dismissed as false-positives. This is because natural selection may affect synonymous codon usage in some genes, leading to codon usage bias [52], [53]. Furthermore, there is increasing evidence that silent mutations may have functional effects either on translational efficiency and accuracy, or on mRNA stability and splicing. Another explanation is that they might carry the footprint of selection on a beneficial allele that is closely linked to the outlier SNP.

In marine environments, environmental factors such as temperature, salinity, pH and dissolved oxygen often interact in complex ways leading to a complicated ‘fitness landscape’. In our study, *C. angulata* and *C. gigas* were sampled from coastal zones, whereas *C. sikamea*, *C. hongkongensis* and *C. ariakensis* were sampled from estuarine zones. Moreover, the five species were collected from 5 sites across 13° of latitude along the coast of China. Therefore, water temperature and salinity may be environmental variations relevant to fitness. The importance of the cytoskeleton in the adaptation to water temperature and salinity is well known [54]–[56]. Major players during cytoskeletal remodeling are rho-GTPases, upstream molecular switches triggering signaling cascades that target cytoskeletal effector proteins to induce morphological change [57]. Another key aspect of the cell stress response is modulation of pathways of energy metabolism [58]. The data presented here reveal that two genes with outlier SNPs (endoglucanase and rho-related GTP-binding protein) are involved in carbohydrate metabolism and GTPase-mediated signal transduction. Furthermore, the ankyrin repeat domain-containing protein 60 may be involved in cytoskeletal motility regulation [59]. Although the genomic scan provides an encouraging result, association genetics and functional studies are ultimately required to confirm that particular loci are involved in responding to environmental variations.

In summary, a total of 57 SNPs from EST sequences in *C. gigas* were developed using HRM method. The study confirmed a high cross-species transferability of the set of 377 EST-SNPs of *C. gigas* tested in four other *Crassostrea* species. Additionally, the current study represents an initial attempt at resolving phylogenetic relationships in *Crassostrea* species, using a large collection of cross-species SNP markers. The NJ analysis revealed two main groups of the five *Crassostrea* species. The first clade included *C. hongkongensis* and *C. ariakensis*. *C. hongkongensis* was a sister species of *C. ariakensis*. This clade was sister to the clade containing *C. sikamea*, *C. angulata* and *C. gigas*. *C. gigas* and *C. angulata* had the closest relationship, with *C. sikamea* being the sister group. Finally, the work, using Fst outlier approaches, presented evidence for adaptive genetic divergence in *Crassostrea* species. Further functional studies are needed to confirm the role of these outlier loci or genome segments in *Crassostrea* species.

## Supporting Information

Table S1 Cross-species amplification of 377 SNPs from *C. gigas* in four other *Crassostrea* species including *C. sikamea*, *C. angulata*, *C. hongkongensis* and *C. ariakensis*.
(XLS)

## References

[1] Hedgecock D (1995) The cupped oyster and the Pacific oyster. In: Thorpe J, Gall G, Lannan J, Nash C eds) Conservation of fish and shellfish resources: managing diversity. pp.115–137.

[2] Harry HW (1985) Synopsis of the supraspecific classification of living oysters (Bivalvia: Gryphaeidae and Ostreidae). Veliger 28: 121–158.

[3] Tack JF, Berghe E, Polk PH (1992) Ecomorphology of *Crassostrea cucullata* (Born, 1778) (Ostreidae) in a mangrove creek (Gazi, Kenya). Hydrobiologia 247: 109–117.

[4] Lapègue S, Boutet I, Leitao A, Heurtebise S, Garcia P, et al (2002) Trans-Atlantic distribution of a mangrove oyster species revealed by 16S mtDNA and karyological analyses. Biol Bull 202: 232–242. 12086994 10.2307/1543473

[5] Banks MA, McGoldrick DJ, Borgeson W, Hedgecock D (1994) Gametic incompatibility and genetic divergence of Pacific and Kumamoto oysters, *Crassostrea gigas* and *C. Sikamea* . Mar Biol 121: 127–135.

[6] Klinbunga S, Khamnamtong N, Tassanakajon A, Puanglarp N, Jarayabhand P (2003) Molecular genetic identification tools for three commercially cultured oysters (*Crassostrea belcheri*, *Crassostrea iredalei* and *Saccostrea cucullata*) in Thailand. Mar Biotechnol 5: 27–36. 12925916 10.1007/s10126-002-0047-4

[7] Boudry P, Heurtebise S, Lapègue S (2003) Mitochondrial and nuclear DNA sequence variation of presumed *Crassostrea gigas* and *Crassostrea angulata* specimens: a new oyster species in Hong Kong? Aquaculture 228: 15–25.

[8] Yu Z, Kong X, Zhang L, Guo X, Xiang J (2003) Taxonomic status of four *Crassostrea oysters* from China as inferred from mitochondrial DNA sequences. J Shellfish Res 22: 31–38.

[9] Reece KS, Cordes JF, Stubbs JB, Hudson KL, Francis EA (2008) Molecular phylogenies help resolve taxonomic confusion with Asian *Crassostrea* oyster species. Mar Biol 153: 709–721.

[10] Wang HY, Guo XM (2008) Identification of *Crassostrea ariakensis* and related oysters by multiplex species-specific PCR. J Shellfish Res 27: 481–487.

[11] Xia J, Yu Z (2009) Identification of seven *Crassostrea oysters* from the South China Sea using PCR–RFLP analysis. J Mollus Stud 75: 139–146.

[12] Ren JF, Liu X, Jiang F, Guo XM, Liu B (2010) Unusual conservation of mitochondrial gene order in *Crassostrea* oysters: evidence for recent speciation in Asia. BMC Evol Biol 10: 394. 21189147 10.1186/1471-2148-10-394 PMC3040558

[13] Wu XY, Xu XD, Yu ZN, Wei ZP, Xia JJ (2010) Comparison of seven *Crassostrea* mitogenomes and phylogenetic analyses. Mol Phylogenet Evol 57: 448–454. 20566320 10.1016/j.ympev.2010.05.029

[14] Yu H, Li Q (2012) Complete mitochondrial DNA sequence of *Crassostrea nippona*: comparative and phylogenomic studies on seven commercial *Crassostrea* species. Mol Biol Rep 39: 999–1009. 21562763 10.1007/s11033-011-0825-z

[15] Hoelzer GA (1997) Inferring phylogenies from mtDNA variation: mitochondrial-gene trees versus nuclear-gene trees revisited. Evolution 51: 622–626. 28565366 10.1111/j.1558-5646.1997.tb02451.x

[16] Hubert S, Higgins B, Borza T, Bowman S (2010) Development of a SNP resource and a genetic linkage map for Atlantic cod (*Gadus morhua*). BMC Genomics 11: 191. 20307277 10.1186/1471-2164-11-191 PMC2846918

[17] Messmer AM, Rondeau EB, Jantzen SG, Lubieniecki KP, Davidson WS, et al (2011) Assessment of population structure in Pacific *Lepeophtheirus salmonis* (Krøyer) using single nucleotide polymorphism and microsatellite genetic markers. Aquaculture 320: 183–192.

[18] Liu ZJ, Cordes JF (2004) DNA marker technologies and their applications in aquaculture genetics. Aquaculture 238: 1–37.

[19] Ollitrault P, Terol J, Garcia-Lor A, Bérard A, Chauveau A, et al (2012) SNP mining in C. clementina BAC end sequences; transferability in the Citrus genus (*Rutaceae*), phylogenetic inferences and perspectives for genetic mapping. BMC Genomics 13: 13. 22233093 10.1186/1471-2164-13-13 PMC3320530

[20] Cunningham C, Hikima J, Jenny MJ, Chapman RW, Fang GC, et al (2006) New resources for marine genomics: bacterial artificial chromosome libraries for the Eastern and Pacific oysters (*Crassostrea virginica* and *C. gigas*). Mar Biotechnol 8: 521–533. 16896533 10.1007/s10126-006-6013-9

[21] Jenny MJ, Chapman RW, Mancia A, Chen YA, McKillen DJ, et al (2007) A cDNA microarray for *Crassostrea virginica* and *C. gigas* . Mar Biotechnol 9: 577–591. 17668266 10.1007/s10126-007-9041-1

[22] Tanguy A, Bierne N, Saavedra C, Pina B, Bachere E, et al (2008) Increasing genomic information in bivalves through new EST collections in four species: development of new genetic markers for environmental studies and genome evolution. Gene 408: 27–36. 18054177 10.1016/j.gene.2007.10.021

[23] Fleury E, Huvet A, Lelong C, Lorgeril J, Boulo V, et al (2009) Generation and analysis of a 29,745 unique expressed sequence tags from the Pacific oyster (*Crassostrea gigas*) assembled into a publicly accessible database: the GigasDatabase. BMC Genomics 10: 34. 19640306 10.1186/1471-2164-10-341 PMC2907693

[24] Jin YL, Kong LF, Yu H, Li Q (2014) Development, inheritance and evaluation of 55 novel single nucleotide polymorphism markers for parentage assignment in the Pacific oyster (*Crassostrea gigas*). Genes Genom 36: 129–141.

[25] Zhong XX, Li Q, Yu H, Kong LF (2013) Development and validation of single-nucleotide polymorphism markers in the Pacific oyster, *Crassostrea gigas*, using high-resolution melting analysis. J World Aquacult Soc 44: 455–465.

[26] Zhong XX, Li Q, Guo X, Yu H, Kong LF (2014) QTL mapping for glycogen content and shell pigmentation in the Pacific oyster *Crassostrea gigas* using microsatellites and SNPs. Aquacult Int doi:10.1007/s10499-014-9789-z

[27] Li Q, Park C, Kijima A (2002) Isolation and characterization of microsatellite loci in the Pacific abalone, *Haliotis discus hannai* . J Shellfish Res 21: 811–815.

[28] Gish W, States DJ (1993) Identification of protein coding regions by database similarity search. Nat Genet 3: 266–272. 8485583 10.1038/ng0393-266

[29] Nei M (1972) Genetic distance between populations. Am Nat 106: 283–292.

[30] Yeh FC, Yang RC, Boyle TBJ, Ye ZH, Mao JX (1999) POPGENE, version 1.32: the user friendly software for population genetic analysis. Molecular Biology and Biotechnology Centre, University of Alberta, Edmonton, AB, Canada.

[31] Tamura K, Peterson D, Peterson N, Stecher G, Nei M, et al (2011) MEGA5: Molecular evolutionary genetics analysis using maximum likelihood, evolutionary distance, and maximum parsimony methods. Mol Biol Evol 28: 2731–2739. 21546353 10.1093/molbev/msr121 PMC3203626

[32] Takezaki N, Nei M, Tamura K (2010) POPTREE2: Software for constructing population trees from allele frequency data and computing other population statistics with Windows-interface. Mol Biol Evol 27: 747–752. 20022889 10.1093/molbev/msp312 PMC2877541

[33] Beaumont MA, Nichols RA (1996) Evaluating loci for use in the genetic analysis of population structure. Proc R Soc Lond B 263: 1619–1626.

[34] De Wit P, Palumbi SR (2013) Transcriptome-wide polymorphisms of red abalone (*Haliotis rufescens*) reveal patterns of gene flow and local adaptation. Mol Ecol 22: 2884–2897. 23106543 10.1111/mec.12081

[35] Milano I, Babbucci M, Cariani A, Atanassova M, Bekkevold D, et al (2014) Outlier SNP markers reveal fine-scale genetic structuring across European hake populations (*Merluccius merluccius*). Mol Ecol 23: 118–135. 24138219 10.1111/mec.12568

[36] Reddy MRK, Rathour R, Kumar N, Katoch P, Sharma TR (2010) Cross-genera legume SSR markers for analysis of genetic diversity in *Lens* species. Plant Breeding 129: 514–518.

[37] Zeid M, Yu JK, Goldowitz I, Dentond ME, Costich DE, et al (2010) Cross-amplification of EST-derived markers among 16 grass species. Field Crop Res 118: 28–35.

[38] Li Q, Liu SK, Kong LF (2009) Microsatellites within genes and ESTs of the Pacific oyster *Crassostrea gigas* and their transferability in five other *Crassostrea* species. Electron J Biotechn 12: 3.

[39] Hedgecock D, Li G, Hubert SK, Buckli A, Ribes V (2004) Widespread null alleles and poor cross-species amplification of microsatellite DNA cloned from the Pacific oyster, *Crassostrea gigas* . J Shellfish Res 23: 379–385.

[40] Boudry P, Heurtebise S, Collet B, Cornette F, Gerard A (1998) Differentiation between populations of the Portuguese oyster, *Crassostrea angulata* (Lamark) and the Pacific oyster, *Crassostrea gigas* (Thunberg), revealed by mtDNA RFLP analysis. J Exp Mar Biol Ecol 226: 279–291.

[41] Lapègue S, Batista FM, Heurtebise S, Yu Z, Boudry P (2004) Evidence for the presence of the Portuguese oyster, *Crassostrea angulata*, in northern China. J Shellfish Res 23: 759–763.

[42] López-Flores I, de la Herrán R, Garrido-Ramos MA, Boudry P, Ruiz-Rejón C, et al (2004) The molecular phylogeny of oysters based on a satellite DNA related to transposons. Gene 339: 181–188. 15363858 10.1016/j.gene.2004.06.049

[43] Imai T, Sakai S (1961) Study of breeding of Japanese oysters, *Crassostrea gigas* . Tohoku Journal of Agricultural 12: 125–171.

[44] Huvet A, Balabaud K, Bierne N, Boudry P (2001) Microsatellite analysis of 6-hour-old embryos reveals no preferential intraspecific fertilization between cupped oysters *Crassostrea gigas* and *Crassostrea angulata* . Mar Biotechnol 3: 448–453. 14961337 10.1007/s10126-001-0017-2

[45] Huvet A, Gerard A, Ledu C, Phelipot P, Heurtebise S, et al (2002) Is fertility of hybrids enough to conclude that the two oysters *Crassostrea gigas* and *Crassostrea angulata* are the same species? Aquat Living Resour 15: 45–52.

[46] Beaumont MA (2005) Adaptation and speciation: what can Fst tell us? Trends Ecol Evol 20: 435–440. 16701414 10.1016/j.tree.2005.05.017

[47] Excoffier L, Hofer T, Foll M (2009) Detecting loci under selection in a hierarchically structured population. Heredity 103: 285–298. 19623208 10.1038/hdy.2009.74

[48] Luikart G, England PR, Tallmon D, Jordan S, Taberlet P (2003) The power and promise of population genomics: from genotyping to genome typing. Nat Rev Genet 4: 981–994. 14631358 10.1038/nrg1226

[49] Li JR, Li HP, Jakobsson M, Li S, Sjödin P, et al (2012) Joint analysis of demography and selection in population genetics: where do we stand and where could we go? Mol Ecol 21: 28–44. 21999307 10.1111/j.1365-294X.2011.05308.x

[50] Storz JF (2005) Using genome scans of DNA polymorphism to infer adaptive population divergence. Mol Ecol 14: 671–688. 15723660 10.1111/j.1365-294X.2005.02437.x

[51] Bierne N, Welch J, Loire E, Bonhomme F, David P (2011) The coupling hypothesis: why genome scans may fail to map local adaptation genes. Mol Ecol 20: 2044–2072. 21476991 10.1111/j.1365-294X.2011.05080.x

[52] Williams EJ, Hurst LD (2000) The proteins of linked genes evolve at similar rates. Nature 407: 900–903. 11057667 10.1038/35038066

[53] Chamary JV, Hurst LD (2004) Similar rates but different modes of sequence evolution in introns and at exonic silent sites in rodents: evidence for selectively driven codon usage. Mol Biol Evol 21: 1014–1023. 15014158 10.1093/molbev/msh087

[54] Evans T, Somero GN (2008) A microarray-based transcriptomic time-course of hyper- and hypoosmotic signaling events in the euryhaline fish *Gillichthys mirabilis*: osmosenors to effectors. J Exp Biol 211: 3636–3649. 18978229 10.1242/jeb.022160

[55] Zhao XL, Yu H, Kong LF, Li Q (2012) Transcriptomic responses to salinity stress in the Pacific Oyster *Crassostrea gigas* . PLoS ONE 7: e46244. 23029449 10.1371/journal.pone.0046244 PMC3459877

[56] Gracey AY, Fraser EJ, Li W, Fang Y, Taylor RR, et al (2004) Coping with cold: an integrative, multitissue analysis of the transcriptome of a poikilothermic vertebrate. P Natl Acad Sci USA 101: 16970–16975. 10.1073/pnas.0403627101 PMC534716 15550548

[57] Di Ciano-Oliveira C, Thirone AC, Szászi K, Kapus A (2006) Osmotic stress and the cytoskeleton: the R(h)ole of Rho-GTPases. Acta Physiol 187: 257–272. 10.1111/j.1748-1716.2006.01535.x 16734763

[58] Kültz D (2005) Molecular and evolutionary basis of the cellular stress response. Annu Rev Physiol 67: 225–257. 15709958 10.1146/annurev.physiol.67.040403.103635

[59] Mosavi LK, Cammett TJ, Desrosiers DC, Peng ZY (2004) The ankyrin repeat as molecular architecture for protein recognition. Protein Sci 13: 1435–1448. 15152081 10.1110/ps.03554604 PMC2279977

